# Systematic Review and Meta‐Analysis of the Transitions, Trajectories, and Associations With Mortality of Frailty in China

**DOI:** 10.1002/hcs2.70112

**Published:** 2026-11-02

**Authors:** Dongfeng Tang, Canlong Wang, Katie Jane Sheehan, Julie Whitney, Aicha Goubar, Salma Ayis, Matthew D. L. O'Connell

**Affiliations:** ^1^ School of Life Course and Population Sciences, Faculty of Life Science and Medicine, King's College London London United Kingdom; ^2^ Bone & Joint Health, Blizard Institute Queen Mary University of London London United Kingdom; ^3^ Department of Clinical Gerontology King's College Hospital NHS Foundation Trust London United Kingdom

**Keywords:** care need, functional decline, health outcome, healthy longevity

## Abstract

Frailty care needs are increasing in China's ageing population, but evidence on frailty progression and outcomes in this context has remained limited. This study aims to synthesize existing evidence on frailty trajectories as well as on the associations between frailty and mortality. English‐language databases (MEDLINE, Embase, Web of Science, PsycINFO, CINAHL) and Chinese databases (China National Knowledge Infrastructure, Wanfang Data, VIP Information) were searched up to October 2024. Random‐effects meta‐analysis was applied to estimate pooled frailty transitions and mortality risks, and frailty trajectories derived by statistical modelling were narratively summarized. Frailty status was stable for 61% (95% confidence interval [CI]: 57%–66%) of individuals, worsened for 29% (95% CI: 24%–34%), and improved for 10% (95% CI: 7%–13%) among Chinese adults aged ≥ 30 years with 2–11 years of follow‐up. Levels or changes of frailty were used to define trajectory patterns. A 1% increase in the frailty index was associated with a 5% higher mortality risk (hazard ratio = 1.05, 95% CI: 1.03–1.06), while frailty was linked to over a twofold higher mortality risk compared with robustness (hazard ratio = 2.34, 95% CI: 1.51–3.63). Frailty is associated with increased mortality risk among Chinese community‐dwelling adults, and progression is not inevitable, with many remaining stable. Distinct subpopulations can be identified by frailty levels or changes. Future research should examine determinants and outcomes of frailty transitions and trajectories to guide targeted interventions.

## Graphical abstract

The review shows frailty progression is not inevitable and distinct subgroups with different trajectories exist. The strong link with mortality underscores the need for routine frailty assessment in community and clinical settings.

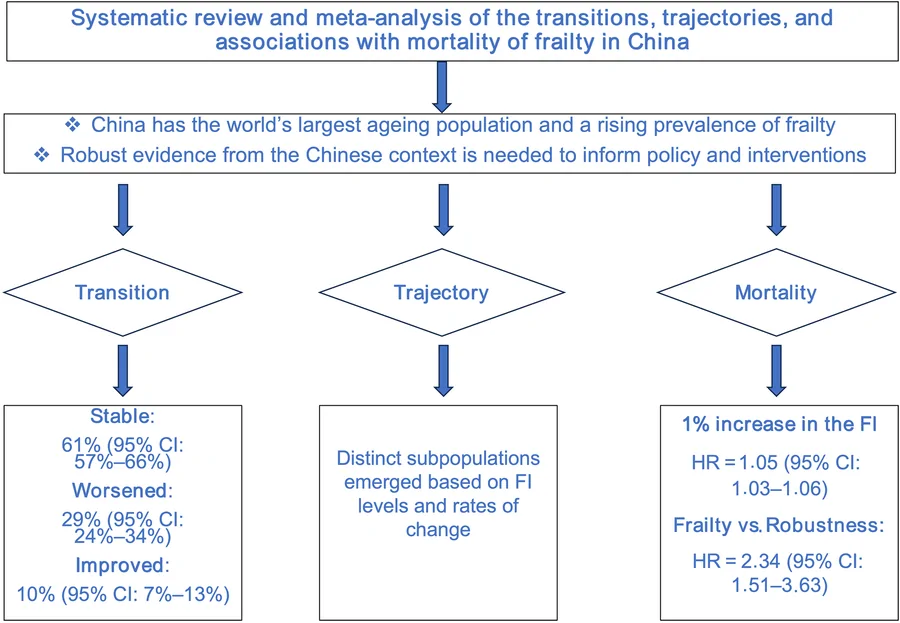

**Abbreviations**
ADL: activity of daily living
BLSA: Beijing Longitudinal Study of Aging
BMI: body mass index
CHARLS: China Health and Retirement Longitudinal Study
CI: confidence interval
CKB: China Kadoorie Biobank
CLHLS: Chinese Longitudinal Healthy Longevity Survey
CNKI: China National Knowledge Infrastructure
DL: DerSimonian and Laird
FI: frailty index
FP: frailty phenotype
FRAIL: fatigue, resistance, ambulation, illnesses, loss of weight
HR: hazard ratio
IQR: interquartile range
OR: odds ratio
RuLAS: Rugao Longevity and Ageing Study
SAGE: Study on Global Ageing and Adult Health
SD: standard deviation
SOF: Study of Osteoporotic Fractures
VIP: VIP Information
WCHAT: West China Health and Aging Trend
WOS: Web of Science

## 1 Background

China has the world's largest ageing population and a rising prevalence of frailty [1, 2]. This trend is driving growing health and social care needs [3]. In response, the government has launched the Healthy China 2030 initiative to promote health through reform and innovation [3]. Yet, robust evidence from the Chinese context is still needed to inform policy and interventions.

Although age is a major risk factor, frailty is not an inevitable outcome of ageing but a dynamic condition with potential for progression and recovery [4, 5]. Therefore, it is imperative to clarify the course of frailty, which helps to predict the growth of the frail population size and inform potential interventions. In China, research has addressed frailty assessment, epidemiology, biomarkers, prevention, and treatment, but evidence on frailty transitions and trajectories remains relatively limited [3]. Several reviews on frailty transitions and trajectories conducted in broader populations have largely relied on narrative synthesis, offered little systematic evaluation of statistical approaches, and focused mainly on the frailty phenotype (FP) or frailty index (FI) [5, 6, 7, 8, 9]. Given the absence of a universally accepted definition, these reviews have overlooked heterogeneity across measures. Moreover, most evidence comes from Western populations, limiting generalizability to China given distinct ethnic, healthcare, and socioeconomic contexts. This underscores the need to synthesize frailty evidence specific to China.

Frailty is a well‐established predictor of mortality, including in Chinese populations [10, 11]. Previous reviews have examined this association in subgroups with hypertension, dialysis, surgery, arthroplasty, or human immunodeficiency virus, consistently linking frailty to increased mortality risk [12, 13, 14, 15, 16]. However, these findings are not generalizable to community‐dwelling adults. A meta‐analysis focused on community‐based Chinese populations is needed to quantify the health impact of frailty and provide context‐specific evidence to guide prevention, intervention, and healthy ageing strategies in China.

This study aims to clarify three questions among the community‐dwelling Chinese adults. First, it seeks to synthesize current evidence to identify the proportions of individuals transitioning from different frailty states. Second, it aims to provide a narrative synthesis of the reported frailty trajectory patterns identified in longitudinal studies. Third, it aims to synthesize the evidence on the association between frailty and mortality, to quantify the prognostic implications of frailty.

## 2 Methods

### 2.1 Data Source and Search Strategy

This study was conducted in accordance with the registered protocol (PROSPERO number: CRD42024604407). A systematic literature search was performed up to October 24, 2024 using both English‐language databases (MEDLINE, Embase, Web of Science, PsycINFO, and CINAHL), and Chinese‐language databases (China National Knowledge Infrastructure, Wanfang Data, and VIP Information). No language restrictions were applied, and studies published in either English or Chinese were eligible for inclusion. Medical Subject Headings and text terms were applied to search for studies. Key terms related to frailty were frail, frailty, frailness, frailty syndrome, pre‐frail, frailty index, asthenia, frail elderly, and accumulated deficit. Key terms about transition or trajectory were trajectory, change, transition, progression, recovery, increase, decrease, worsening, improving, pattern, profile, and trend. Mortality key terms were all‐cause mortality, mortality, death, survival, survival rate, survival analysis, and Kaplan–Meier estimate. Key terms including China, Chinese mainland, and Chinese were also used. Apart from studies themed at transitions or trajectories of frailty and associations between frailty and mortality, prevalence of frailty was also included in the initial protocol. Therefore, key terms regarding frailty prevalence were also included in the initial search, and then excluded in the study selection phase. The detailed search strategies are described in Supporting Information S1: Data S1. After protocol submission and initial searches, it became clear the evidence on the prevalence of frailty in China is well established and has been previously synthesized with limited recent updates [17, 18], this topic was excluded from further review.

### 2.2 Study Selection

Inclusion criteria were: (1) General population of community‐dwelling adults aged ≥ 18 years and Chinese people living in Chinese mainland. (2) Studies in which frailty was defined by validated tools such as FP, FI, and the clinical frailty scale. (3) Reporting formally modelled frailty trajectories using statistical approach or proportions of individuals with different frailty changes. (4) Reporting associations of frailty with mortality. Studies were excluded if they (1) were studies about prevalence of frailty which were included based on the initial protocol. (2) were not eligible in age or, non‐Chinese or Chinese living outside of Chinese mainland, or not community‐dwelling. (3) used not validated measurements. (4) cognitive frailty or social frailty. (5) were narrative without statistical modelling or calculation of proportions of different frailty changes. (6) were intervention studies, reviews, editorials, case studies/case series, or qualitative studies. Studies using repeated cohorts were not excluded, aiming to narratively present results across frailty measurements, follow‐up duration and other sources of heterogeneity.

Screening for titles, abstracts and full texts of studies and selection were completed in duplicate using the online platform Covidence by two independent reviewers. The disagreements between two reviewers were resolved by discussion, and further third reviewer consultation was employed when the consensus failed to be achieved.

### 2.3 Data Extraction and Study Quality Assessment

Data from eligible studies were extracted by one investigator and independently confirmed by another investigator. Extracted study details were first author, publication year, study/cohort name, urban or rural area, sample size, age characteristics, sex composition, frailty measurement, duration of follow‐up, and language. For frailty trajectory studies, baseline frailty status, and proportions of different frailty status transitions or modelled trajectories were extracted. Mortality effects estimate type, comparator for hazard ratio (HR) or odds ratio (OR), effect size, and covariates were obtained for mortality studies.

To assess the methodological quality of included studies, an adapted Newcastle‐Ottawa Scale for systematic reviews of frailty studies was used [19]. Three criteria, including representative, ascertainment of exposure frailty, and non‐respondents, which could be applied to frailty trajectory studies, were used. Seven additional questions were applied to assess frailty mortality risk studies, including selection of a non‐frail comparison group, outcome not present at start, controls for age and sex, controls for other factors, outcome assessment, length of follow‐up, and adequacy of follow‐up. The details of the scale have been published previously [19].

### 2.4 Statistical Analysis

Different proportions of frailty transitions were defined as: (1) stable, frailty status remains in robust, or prefrail or frail. (2) worsened, frailty status transitioned from robust to prefrail or frail or prefrail to frail. (3) improved, from frail to prefrail or robust, or from prefrail to robust. HR of mortality for frailty were presented in two different ways: (1) Mortality HR for 1% increase in FI. (2) Mortality HR for frail participants compared to robust. To present the heterogeneity across studies, studies on frailty transitions were grouped based on frailty measurement, age (≥ 60 years, ≥ 45 years, and ≥ 30 years), follow‐up duration (< 5, 5–9, and ≥ 10 years), and baseline frailty status of population (general, robust and prefrail). For studies regarding mortality risk, they were categorized by frailty measurement, comparator of HR (robust, 1% increase in FI, and non‐frail), age, and follow‐up duration. Meta‐analyses for studies within certain subgroups were conducted when the following criteria were met: (1) having more than one study, and (2) having more than one cohort. The overall results of different proportions of frailty transition and mortality HRs for 1% increase in FI score and being frail were also pooled by meta‐analyses. When conducting meta‐analysis, random‐effects models were used, and where there were multiple reports from the same cohorts, only one study from each cohort was included. In such case, the study was selected based on the following priority: the most recent sample, largest sample size, and longest follow‐up duration. Other studies using the same cohort were excluded. Heterogeneity across studies was quantified by Cochrane's *Q* test and/or *I* ^2^ statistics. If *I* ^2^ statistic reached 50% or more, and/or a *p* value was less than 0.10, the heterogeneity was regarded as significant. Finally, publication bias was assessed by funnel plots. To explore the disparities of three frailty transition patterns and mortality risk, meta‐analyses were repeated stratified by frailty measurement, area (urban or rural), age, and follow‐up duration. For studies reporting trajectories of frailty modelled by statistical approach, meta‐analysis was not feasible and therefore narrative analysis was conducted instead.

All analyses were conducted by StataMP 18.0 (StataCorp LP, College Station, United States).

## 3 Results

### 3.1 Search Results and Study Characteristics

A total of 13,598 publications were obtained from the initial literature research. Following de‐duplication, 6962 studies underwent title and abstract screening. From these, 267 studies underwent full‐text selection, with 196 excluded on outcome, 14 on population, 15 on study type, five on full text not available (Figure 1). Therefore, 37 studies were included, of which 14 explored proportions of frailty state transitions, 10 identified frailty trajectory patterns [20, 21, 22, 23, 24, 25, 26, 27, 28, 29, 30, 31, 32, 33, 34, 35, 36, 37, 38, 39, 40, 41, 42, 43], and 13 [11, 44, 45, 46, 47, 48, 49, 50, 51, 52, 53, 54, 55] explored the mortality risk of frailty.

**Figure 1:**
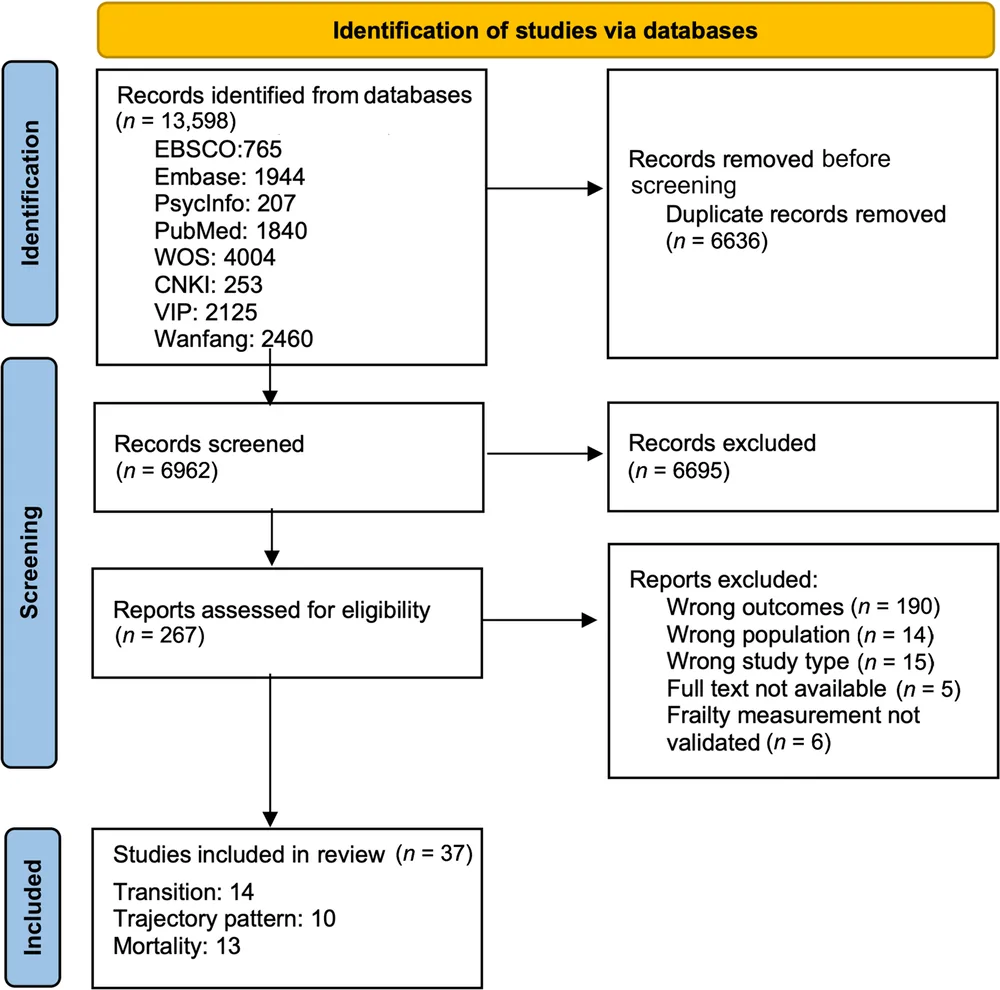
Flowchart of studies inclusion and exclusion. CNKI, China National Knowledge Infrastructure; VIP, VIP Information; Wanfang, Wanfang Data; WOS, Web of Science.

Table 1 presents the characteristics of studies investigating frailty transitions or trajectory patterns published between 2018 and 2024, with sample sizes ranging from 196 to 23,847. Nine cohorts were employed by 24 studies including West China Health and Aging Trend study, a continuing care retirement community‐based survey in Beijing, China Health and Retirement Longitudinal Study (CHARLS), China Kadoorie Biobank, the Chinese Longitudinal Healthy Longevity Survey, a community‐based survey in Shanghai, an urban community‐based aging follow‐up study in Dujiangyan City, and Study on Global Ageing and Adult Health, MJ Health Screening Center. Participants were followed up between two to 16 years and different measurements of frailty were used, consisting of FI, FP, the fatigue, resistance, ambulation, illnesses, loss of weight (FRAIL) scale, and the Study of Osteoporotic Fractures frailty index.

**Table 1:** Characteristics of studies for frailty states transitions and trajectory patterns of frailty.

Study	Urban/rural	Cohort	Sample size	Age characteristics (years)	Sex composition	Frailty measurement	Duration of follow‐up (years)	Result type	Baseline status of population	Result	Language
Wang et al. [20]	Mixed	CLHLS	2805	Median (IQR): 80 (73–87)	Male: 48.84% Female: 51.16%	The SOF index	4 (2014–2018)	Portion	General	Stable: 67% Worsen: 18% Improve: 15%	English
Duan et al. [21]	Mixed	CHARLS	5411	Mean ± SD: 57.77 ± 8.73	Male: 41.32% Female: 58.68%	FI	7 (2011–2018)	Portion	Pre‐frail	Stable: 53.8% Worsen: 31.5% Improve: 14.7%	English
Jia et al. [22]	Mixed	WCHAT	808	Mean ± SD: 67.8 ± 5.1	Male: 40.5% Female: 59.5%,	FP	3 (2018–2021)	Portion	General	Kept not‐frail or improved (transitions from frail to not‐frail): 93.2% Kept frail or worsened (transitions from not‐frail to frail): 6.8%	English
Ye et al. [23]	Mixed	Community‐based survey in Shanghai	3988	Mean ± SD: 69.38 ± 7.06	Male: 43.5% Female: 56.5%	FI	2 (2015–2017)	Portion	General	Stable: 70.0% Worsen: 22.2% Improve: 7.8%	English
He et al. [24]	Mixed	CHARLS	7116	Mean ± SD: 57.4 ± 8.5	Male: 51.4% Female: 48.6%	FI	5 (2013–2018)	Portion	General	Stable: 66.4% Worsen: 18.0% Improve: 15.6%	English
Bi et al. [25]	Mixed	CLHLS	1645	Mean ± SD: 74.73 ± 7.45	Male: 48% Female: 52%	FI	10 (2008–2018)	Modelled trajectories	General	Gradual increase in FI: 81.5% Swiftly expanding FI: 18.5%	English
Yang et al. [26]	Mixed	CLHLS	2299	76.9% were aged 65–79 years; remaining individuals aged ≥ 80 years	Male: 48.5% Female: 51.5%	FI	16 (2002–2018)	Modelled trajectories	Not frail	Non‐frail: 56.5% Moderate progressive: 32.1% High progressive trajectories: 11.4%	English
Lin et al. [27]	Mixed	CLHLS	6713	Mean ± SD: 79.9 ± 10.2 in level of daily participation; 86.32 ± 9.30 in often level of social participation; 90.80 ± 8.97 in occasionally level of social participation.	Male: 49.0% Female: 51.0%	FI	10 (2008–2018)	Modelled trajectories	Not frail	Stable development: 73.4% Rapid growth: 26.6%	English
Yang et al. [28]	Urban	Urban community‐based aging follow‐up study in Dujiangyan City	507	Mean ± SD: 72.99 ± 6.56	Male: 40.2% Female: 59.8%	FRAIL scale	3 (2014–2017)	Portion	General	Stable: 54.5% Worsen: 40.4% Improve: 5.1%	Chinese
Du et al. [29]	Mixed	CLHLS	2268	Mean ± SD: 75.5 ± 8.03	Male: 46.3% Female: 53.7%	FRAIL scale and FI	10 (2008–2018)	Modelled trajectories	General	FRAIL: No‐frailty: 58.82% Increasing‐frailty: 17.02% Worsened‐frailty: 12.17% Improved‐frailty: 11.99% FI: Low‐stable: 81.35% Medium‐stable: 8.29% Low‐rapid: 10.36%	English
Guo et al. [30]	Mixed	CHARLS	8993	Mean ± SD: 61.3 ± 7.8	Male: 45.2% Female: 54.8%	FI	7 (2011–2018)	Modelled trajectories	General	Low and stable trajectory: 56.8% Moderate and increasing trajectory: 34.4% High and increasing trajectory: 8.8%	English
Guo et al. [31]	Mixed	SAGE	4303	46.9% aged 50–59 years	Male: 49.1% Female: 50.9%	FI	8 (2010–2018)	Modelled trajectories	General	Low and stable trajectory: 75.9% Moderate and increasing trajectory: 19.1% High and increasing trajectory: 5.0%	English
Liu et al. [32]	Mixed	CLHLS	11,165	Mean: 82.6	Male: 48.0% Female: 52.0%	FI	3 (2002–2005)	Portion	General	Stable: 37.6% Worsen: 53.1% Improve: 9.3%	English
Chu et al. [33]	Mixed	CHARLS	2961	Mean ± SD: 67.0 ± 5.52	Male: 51.0% Female: 49.0%	FP	4 (2011–2015)	Portion	General	Stable: 59% Worsen: 21% Improve: 20%	English
Wu et al. [34]	Mixed	CLHLS	4083	Mean: 77.0	Male: 48.0% Female: 52.0%	FI	16 (2002–2018)	Modelled trajectories	General	Stable‐growth: 82.54% Rapid‐growth: 17.46%	English
Sha et al. [35]	Mixed	CLHLS	3‐year follow‐up: 5746; 6‐year period: 3548	Mean ± SD: 82.6 ± 11.0 (3 years follow‐up) and 79.1 ± 10.1 (6 years follow‐up)	Male: 45.3% (3 years follow‐up) Female: 54.7% (3 years follow‐up) Male: 46.6% (6 years follow‐up) Female: 53.4% (6 years follow‐up)	FRAIL scale	3 (2008–2011) and 6 (2008–2014)	Portion	General	3 years: Stable: 50.6% Worsen: 28.7% Improve (20.6%) 6 years: Stable: 49.1% Worsen: 31.9% Improve: 19.1%)	English
Zhu et al. [36]	Mixed	CKB	23,847	Mean: 51.2	Male: 38.3% Female: 61.7%	FI	median follow up: 8	Portion	Not frail	Stable: 60.9% Worsen: 31.7% Improve: 7.4%	English
Pan et al. [37]	Mixed	CLHLS	1316	Mean ± SD: 85.84 ± 11.43	Male: 43.26% Female: 56.74%	FI	13 (2005–2018)	Modelled trajectories	General	Low frailty: 59.00% Middle frailty: 34.07% High frailty: 6.93%	English
Sha et al. [38]	Mixed	CHARLS	4618	Age groups: 60–64 (40%), 65–74 (46%), ≥ 75 (14%)	Male: 52.0% Female: 48.0%	FP	4 (2011–2015)	Portion	Not frail	Stable: 56.9% Worsen: 23.8% Improve: 19.4%	English
Sheng et al. [39]	Mixed	CHARLS	5367	Median (IQR): 66 (62–71)	Male: 51.4% Female: 48.6%	FRAIL scale	2 (2013–2015)	Portion	General	Stable: 57.5% Worsen: 24.7% Improve: 17.8%	English
Liu et al. [40]	Urban	A continuing care retirement community‐based survey in Beijing	196	Mean ± SD: 83.7 ± 4.4	Male: 40.8% Female: 59.2%	FRAIL scale	2 (2018–2020)	Portion	General	Stable: 53.1% Worsen: 38.3% Improve: 8.6%	English
Fan et al. [41]	Urban	MJ Health Screening Center, Beijing	13,689	Mean ± SD: 42.3 ± 9.2	Male: 55.7% Female: 44.3%	FI	Follow‐up mean ± SD: 5.7 ± 1.9, range from 3–11	Portion	General	Stable: 67.3% Worsen: 21.2% Improve: 12.5%	English
Zhuo et al. [42]	Mixed	CLHLS	7488	Mean ± SD: 76.92 ± 9.0 (stable health group); 85.53 ± 9.7 (progressive into frailty group); 89.76 ± 8.9 (stable frailty group)	Male: 45.95% Female: 54.05%	FI	16 (2002–2018)	Modelled trajectories	General	Stable health: 62.22% Progressive into frailty: 25.55% Stable frailty: 12.20%	Chinese
Chen [43]	Mixed	CLHLS	2443	Aged over 65 years	Not reported	FI	9 (2005–2014)	Modelled trajectories	General	Stable health: 24.2% Stable progressive: 35.0% Rapid progressive: 40.8%	Chinese

Abbreviations: CHARLS, China Health and Retirement Longitudinal Study; CKB, China Kadoorie Biobank; CLHLS, Chinese Longitudinal Healthy Longevity Survey; FI, frailty index; FP, frailty phenotype; FRAIL, fatigue, resistance, ambulation, illnesses, loss of weight; IQR, interquartile range; SAGE, Global Ageing and Adult Health Research; SD, standard deviation; SOF, Study of Osteoporotic Fractures; WCHAT, West China Health and Aging Trend Study.

Table 2 summarizes the characteristics of studies investigating frailty mortality risk, published during 2016–2024. Number of participants ranges from 1246 to 512,723, recruiting from cohorts including Beijing Longitudinal Study of Aging, Health Status and Fall Status Follow‐up Survey Database, CHARLS, China Kadoorie Biobank, the Chinese Longitudinal Healthy Longevity Survey, Rugao Longevity and Ageing Study, and Study on Global Ageing and Adult Health. FI and FP were used as measurements for frailty and HR or OR for mortality of 1% increase in FI and being frail were estimated. Quality of included studies was presented in Supporting Information S1: Table S1.

**Table 2:** Characteristics of mortality risk studies.

Study	Urban/rural	Cohort	Sample size	Age characteristics (years)	Sex composition	Frailty measurement	Duration of follow‐up (years)	Mortality effect estimate type	Comparator	Effect (95% CI)	Covariates	Language
Wang et al. [44]	Mixed	BLSA	1257	Mean ± SD: 69.2 ± 8.1, ranging 55–97	Male: 43.8% Female: 56.2%	FI	5 (2009–2014)	HR	1% increase in FI	1.04 (1.03–1.05)	Age and sex	English
Shi et al. [45]	Urban	Health Status and Fall Status Follow‐up Survey Database	1246	Mean ± SD: 72.05 ± 4.52, ranging 60–101	Male: 41.7% Female: 58.3%	FI	11 (2009–2020)	HR	1% increase in FI	1.145 (1.07–1.22)	Age, sex, education, marital status, employment	English
Cao et al. [46]	Mixed	CHARLS	3929	Mean ± SD: 67.4 ± 6.3	Male: 53.5% Female: 46.5%	FI	6 (2011/12–2018)	OR	Robust	OR = 1.82 (1.41–2.35)	Age, sex, education, residence, number of chronic diseases	English
Sun et al. [47]	Mixed	CHARLS	5630	Mean ± SD: 74.29 ± 6.21	Male: 50.6% Female: 49.4%	Frailty Phenotype	8 (2011–2018)	OR	Robust	2013: OR = 2.59 (1.46–4.59) 2015: OR = 1.60 (1.26–2.04) 2018: OR = 1.58 (1.31–1.90)	Age, sex, education, marriage status, residence, smoking, alcohol drinking	Chinese
Wu et al. [48]	Mixed	CHARLS	5300	Mean ± SD: 67.8 ± 6.5	Male: 48.9%, Female: 51.1%	Frailty Phenotype	Median: 6.22 (2011–2018)	HR	Robust	2.38	Age, sex, education, urban residence, marital status, depression, cognitive status	English
Fan et al. [49]	Mixed	CKB	23,615	Mean ± SD: 59.2 ± 10.0, ranging 38–87	Male: 38.3% Female: 61.7%	Frailty Phenotype; FI	Median ± SD: 3.9 ± 0.5	HR	Robust; 1% increase in FI	Compared to robust: HR = 2.90 (2.25–3.73) 1% increase in FI: HR = 1.06 (1.05–1.08)	Age, education, smoking, alcohol, diet (vegetable/fruit/meat intake)	Chinese
Fan et al. [11]	Mixed	CKB	512,723	Mean ± SD: 52.0 ± 10.7	Male: 41.0%, Female: 59.0%	FI	Median 10.8 (IQR 10.2–13.1)	HR	Robust; 1% increase in FI	1% increase in FI: HR = 1.053 (1.052–1.055) Compared to robust: HR = 3.08 (2.98–3.18)	Age, sex, region, education, smoking, alcohol, fruit/vegetable/red meat intake, family history (heart attack, stroke, cancer)	English
Zhao et al. [50]	Mixed	CLHLS	8642	Median, ± SD: 85.6, ± 11.3, ranging 50–114	Male: 45.2%, Female: 54.8%	FI	7 (2011–2018)	HR	Robust; 1% increase in FI	1% increase in FI: 1.037 (1.035–1.039) robust: 1.820 (1.640–2.021)	Age, sex, marital status, education, living conditions, residence	English
She et al. [51]	Mixed	CLHLS	5134	Mean ± SD: 87.5 ± 11.4, ranging 65– > 100	Male: 42.2%, Female: 57.8%	Frailty Phenotype	4 (2014–2018)	HR	Robust	1.47 (1.36–1.59)	Age, sex, education, economic status, urban/rural, smoking, drinking, ADL	English
Shi et al. [52]	Urban	Health Status and Fall Status Follow‐up Survey Database	1301	Mean ± SD: 71.02 ± 5.51	Male: 42.7%, Female: 57.3%	FI	10 (2005–2015)	HR	1% increase in FI	1.143 (1.034–1.248)	Age, sex, education, marital status, occupation status	Chinese
Shi et al. [53]	Rural	RuLAS	1788	Mean ± SD: 75.4 ± 3.9	Male 46.4%, Female 53.6%	Frailty Phenotype; FI	3 (2014–2017)	HR	Robust	FP: 2.24 (1.31–3.83) FI: 2.31 (1.16–4.60)	Age, sex, marital status, education, smoking, drinking, BMI, diabetes, hypertension, cognitive function	English
Gu et al. [54]	Mixed	CLHLS	13,731	High proportions of participants aged ≥ 80	Male: 42.7% Female: 57.3%	FI	3 (2008/2009 to 2011/2012)	HR	1% increase in FI	1.027 (1.025–1.029)	Age, sex, ethnicity, marital status, co‐residence, smoking, regular exercise	English
Sun et al. [55]	Urban	SAGE	7978	Mean ± SD: 62.6 ± 11.5, ranging 45–99	Male: 46.6% Female: 53.4%	FI	Mean ± SD: 11.3 ± 1.8	HR	Non‐frail (robust and prefrail)	1.42 (1.21–1.66)	Age, residence, marriage status, education, household economic status, smoking, alcohol drinking, fruits and vegetables intake, social participation	Chinese

Abbreviations: ADL, activities of daily living; BLSA, Beijing Longitudinal Study of Aging; BMI, body mass index; CHARLS, China Health and Retirement Longitudinal Study; CI, confidence interval; CKB, China Kadoorie Biobank; CLHLS, Chinese Longitudinal Healthy Longevity Survey; FI, frailty index; FP, frailty phenotype; HR, hazard ratio; IQR, interquartile range; OR, odds ratio; RuLAS, Rugao Longevity and Ageing Study; SAGE, Global Ageing and Adult Health Research; SD, standard deviation.

### 3.2 Transitions of Frailty

Supporting Information S1: Figure S1 summarizes the results of the studies which explored the proportions of people transitioning between different states of frailty.

For studies using FI [21, 24, 36, 41], the proportion of individuals with stable, worsened and improved frailty status among middle‐aged and older populations ranged from 54%–67%, 18%–32%, and 7%–16%, respectively. For the population aged 60 years and above with follow‐up less than 5 years, the pooled proportions were 54% (*I* ^2^ = 99.9%, *p* < 0.001) for stable status, 25% (*I* ^2^ = 99.9%, *p* < 0.001) for worsened status, and 9% (*I* ^2^ = 99.9%, *p* < 0.001) for improved status. Among studies using the FRAIL scale [28, 35, 39, 40], the corresponding ranges for stable, worsened, and improved frailty status were 49%–58%, 29%–53%, and 5%–21%, respectively. Specifically, for those studies with follow‐up durations under 5 years [28, 35, 39, 40], the pooled estimates were 54% (*I* ^2^ = 94.4%, *p* < 0.001) for stable status, 22% (*I* ^2^ = 94.4%, *p* < 0.001) for worsened status, and 13% (*I* ^2^ = 98.2%, *p* < 0.001) for improved status. Additionally, two studies using the FP and one using the Study of Osteoporotic Fractures frailty index reported stable frailty status proportions of 59%, 57%, and 67%, respectively [20, 33, 38]. The corresponding proportions for worsened frailty were 21%, 24%, and 18%, while those for improved frailty were 20%, 19%, and 15%, respectively. Of note, one study was not included in Supporting Information S1: Figure S1 because it categorized frailty transitions differently. Specifically, it reported 93.2% of participants as remaining non‐frail or improved (transitioned from frail to non‐frail) and 6.8% as remaining frail or worsened (transitioned from non‐frail to frail) [22].

Figure 2 presents the random‐effect meta‐analysis of seven cohorts which reported the proportion of individuals whose frailty status changed during the study period [20, 21, 23, 28, 36, 40, 41]. For Chinese adults aged 30 years and above with a follow‐up from 2 to 11 years, frailty status was stable for 61% (95% confidence interval [CI]: 57%–66%, *I* ^2^ = 98.6%, *p* < 0.001) of individuals, worsened for 29% (95% CI: 24%–34%, *I* ^2^ = 99.2%, *p* < 0.001), and improved for 10% (95% CI: 7%–13%, *I* ^2^ = 98.6%, *p* < 0.001). Funnel plots are presented in Supporting Information S1: Figure S2, and no significant publication bias was observed. Subgroup analysis by measurement, area, age, and follow‐up duration is presented in Supporting Information S1: Table S2. The results remained consistent with the main analyses. Individuals with stable frailty status accounted for the largest proportion (ranging 54%–64%), followed by those with worsened frailty status (26%–33%). The smallest proportion was observed among individuals who showed improvement in frailty, ranging from 9% to 13%.

**Figure 2:**
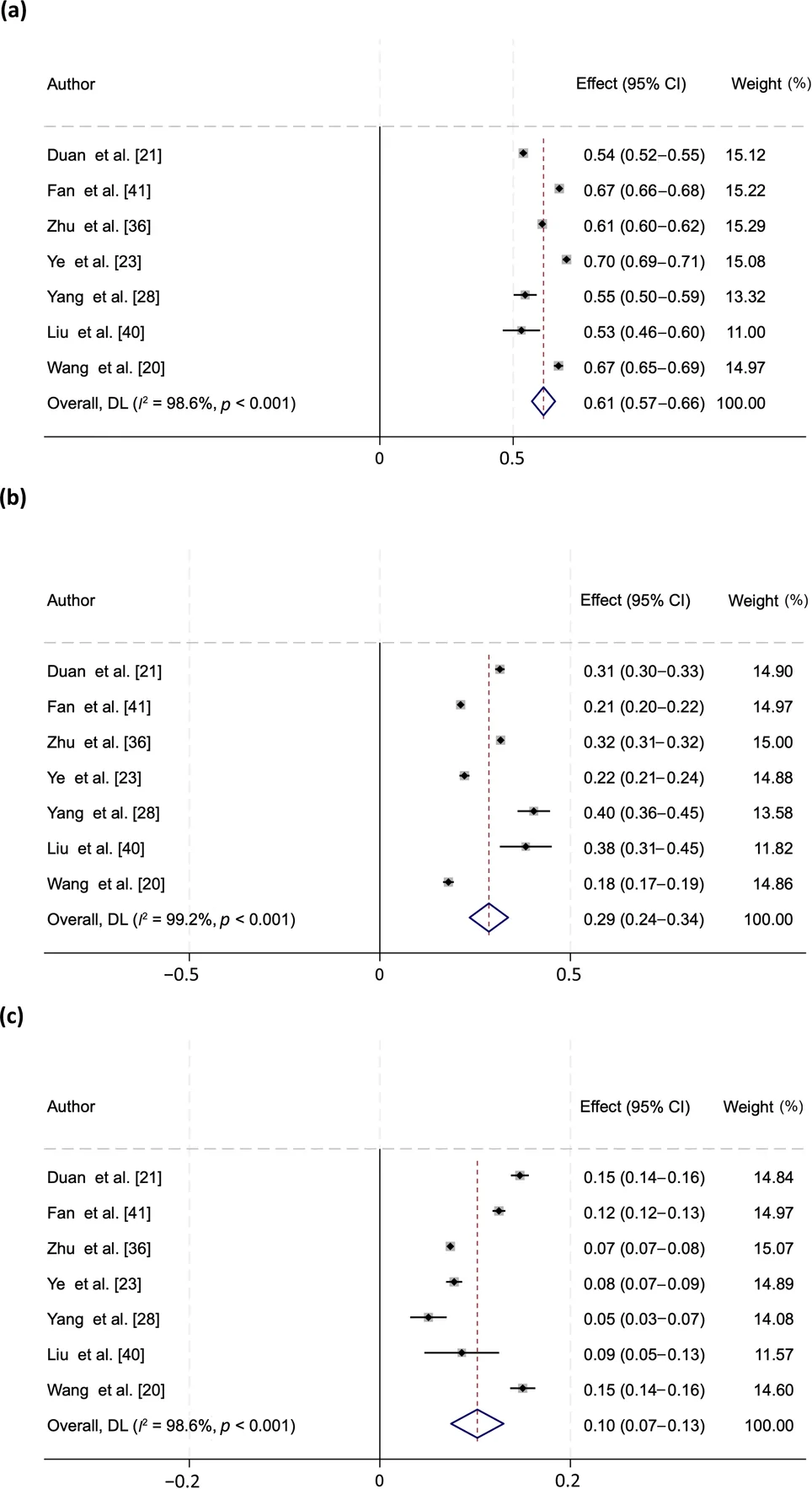
Meta‐analysis for proportions of different frailty status transitions. (a) The proportion of individuals with stable frailty status. (b) The proportion of individuals with worsened frailty status. (c) The proportion of individuals with improved frailty status. Weights are from random‐effects model. CI, confidence interval; DL, DerSimonian and Laird.

### 3.3 Trajectory Patterns of Frailty

Table 3 summarizes the reported frailty trajectory patterns. Ten studies from three cohorts were included with different trajectory patterns defined [25, 26, 27, 29, 30, 31, 34, 37, 42, 43]. Except for one study using FRAIL, all studies explored frailty trajectories measured by FI.

**Table 3:** Defined trajectory patterns of frailty from different studies.

Study	Cohort	Sample size	Age (years)	Frailty measurement	Follow‐up duration (years)	Baseline status of participants	Result
Chen [43]	CLHLS	2443	Older people (≥ 65)	FI	9 (2005–2014)	General	Stable health: 24.2%
							Stable progressive: 35.0%
							Rapid progressive: 40.8%
Bi et al. [25]	CLHLS	1645	Older people (≥ 65)	FI	10 (2008–2018)	General	Gradual increase in FI: 81.5%
							Swiftly expanding FI: 18.5%
Du et al. [29]	CLHLS	2268	Older people (≥ 65)	FRAIL	10 (2008–2018)	General	No‐frailty: 58.8%
							Increasing‐frailty: 17.0%
							Worsened‐frailty: 12.2%
							Improved‐frailty: 12.0%
Du et al. [29]	CLHLS	2268	Older people (≥ 65)	FI	10 (2008–2018)	General	Low‐stable: 81.4%
							Medium‐stable: 8.3%
							Low‐rapid: 10.4%
Wu et al. [34]	CLHLS	4083	Older people (≥ 65)	FI	16 (2002–2018)	General	Stable‐growth: 82.5%
							Rapid‐growth: 17.5%
Pan et al. [37]	CLHLS	1316	Older people (≥ 65)	FI	13 (2005–2018)	General	Low frailty: 59.0%
							Middle frailty: 34.1%
							High frailty: 6.9%
Zhuo et al. [42]	CLHLS	7488	Older people (≥ 65)	FI	16 (2002–2018)	General	Stable health: 62.2%
							Progressive into frailty: 25.6%
							Stable frailty: 12.2%
Lin et al. [27]	CLHLS	6713	Older people (≥ 65)	FI	10 (2008–2018)	Not frail	Stable development: 73.4%
							Rapid growth: 26.6%
Yang et al. [26]	CLHLS	2299	Older people (≥ 65)	FI	16 (2002–2018)	Not frail	Non‐frail: 56.5%
							Moderate progressive: 32.1%
							High progressive: 11.4%
Guo et al. [30]	CHARLS	8993	Middle‐aged and older people (≥ 50)	FI	7 (2011–2018)	General	Low and stable: 56.8%
							Moderate and increasing: 34.4%
							High and increasing: 8.8%
Guo et al. [31]	SAGE	4303	Middle‐aged and older people (≥ 50)	FI	8 (2010–2018)	General	Low and stable: 75.9%
							Moderate and increasing: 19.1%
							High and increasing: 5.0%

Abbreviations: CHARLS, China Health and Retirement Longitudinal Study; CLHLS, Chinese Longitudinal Healthy Longevity Survey; FI, frailty index; FRAIL, fatigue, resistance, ambulation, illnesses, loss of weight; SAGE, Global Ageing and Adult Health Research.

Two studies defined trajectories according to frailty transitions. In Zhuo et al.'s study, 62.2% individuals had stable health, 25.6% and 12.2% were classified as “progressive into frailty” and “stable frailty”, respectively [42]. Similarly, Du et al. reported 58.8% as “no‐frailty” trajectory, 17.0% as “increasing‐frailty”, 12.2% as “worsened‐frailty”, and 12.0% as “improved‐frailty” [29]. Six studies defined trajectories according to the rate of change in the FI score. Among them, three studies modelled trajectories into two groups: rapid increasing and slow increasing [25, 27, 34]. The proportion of individuals in the slow increase group ranged from 73.4% to 82.5%, consistently higher than those in the rapid increase group (17.5%–26.6%). The other three studies defined three levels of progression: (1) low level: “stable health” (24.2%) or “low frailty” (59.0%) or “non‐frail” (56.5%), (2) middle level: “stable progressive” (35.0%) or “middle frailty” (34.1%) or “moderate progressive” (32.1%), (3) high level: “rapid progressive” (40.8%) or “high frailty” (6.9%) or “high progressive” (11.4%) [26, 37, 43]. Finally, three studies defined trajectories according to the level and change of FI score. Two of these studies defined three trajectories: “low and stable” (56.8%–75.9%), “moderate and increasing” (19.1%–34.4%), and “high and increasing” (5.0%–8.8%) [30, 31]. Another study classified trajectories as “low‐stable” (81.4%), “medium‐stable” (8.3%), and “low‐rapid” (10.4%) [29].

### 3.4 Mortality Risk Associated With Frailty

Supporting Information S1: Figure S3 summarizes studies on the association of frailty with mortality. A 1% increase in FI was associated with a mortality HR of 1.03–1.15. For studies among the population aged 30 years and older, and followed up 5–9 years, the HRs of mortality for 1% increase in FI were pooled as 1.04 (95% CI: 1.04–1.04, *I* ^2^ = 0.0%, *p* = 0.564) [44, 50]. The HRs of mortality for being frail compared to robust ranged from 1.47 to 3.08. For studies using FP as the measurement, among population aged 60 years and older with follow‐up less than 5 years [51, 53], the risk of mortality for being frail compared to robust was pooled as 1.66 (95% CI: 1.14–2.42, *I* ^2^ = 56.9%, *p* = 0.128). When compared to a non‐frail population, one study with mean follow‐up duration of 11.3 years reported frail individuals were associated with a mortality HR of 1.42 (95% CI: 1.21–1.66) among the population aged 45 years and older [55]. There were also two studies using CHARLS estimating ORs for mortality for frail compared to robust participants to be from 1.58 (95% CI: 1.31–1.90) to 2.59 (95% CI: 1.46–4.59) [46, 47].

Figure 3 presents the results of meta‐analysis for mortality risk of frailty. 1% increase in FI was associated with a mortality HR of 1.05 (95% CI: 1.03–1.06, *I* ^2^ = 98.2%, *p* < 0.001) for Chinese adults aged 30 years and above with a follow‐up between five and 13 years [11, 44, 45, 50]. Supporting Information S1: Figure S4 presents the publication bias. Using three cohorts [11, 50, 53], the pooled risk of mortality for being frail compared to robust was estimated as 2.34 (95% CI: 1.51–3.63, *I* ^2^ = 97.8%, *p* < 0.001) among Chinese adults aged 30 years and above with a follow‐up between 3 years and 13 years. The corresponding publication bias is presented in Supporting Information S1: Figure S5. Supporting Information S1: Table S3 presents the associations of frailty with mortality in different subgroups of measurement, area, age and follow‐up durations. Overall, frailty was consistently associated with higher mortality risks. The HR for 1% increase in FI among individuals aged 60 years and above was pooled as 1.08 (95% CI: 0.97–1.20, *I* ^2^ = 90.2%, *p* = 0.001). This risk was more pronounced in studies with follow‐up ≥ 10 years (HR = 1.09, 95% CI:1.01–1.18, *I* ^2^ = 83.5%, *p* = 0.014), compared to those with shorter follow‐up years (HR_5–9 years_ = 1.04, HR_< 5years_ = 1.04). When compared to robust measured by FI, the pooled mortality HR for being frail (2.36, 95% CI: 1.50–3.71, *I* ^2^ = 97.8%, *p* < 0.001) was higher than corresponding pooled HR among studies measured by FP (2.09, 95%CI: 1.24–3.53, *I* ^2^ = 92.6%, *p* < 0.001). In the studies with individuals aged 60 years and above, the pooled HR of mortality for being frail compared to robust was 1.66 (95% CI: 1.14–2.42, *I* ^2^ = 56.9%, *p* = 0.128). The corresponding pooled HR from studies with less than 5 years follow‐up was 2.09 (95% CI: 1.24–3.53, *I* ^2^ = 92.6%, *p* < 0.001).

**Figure 3:**
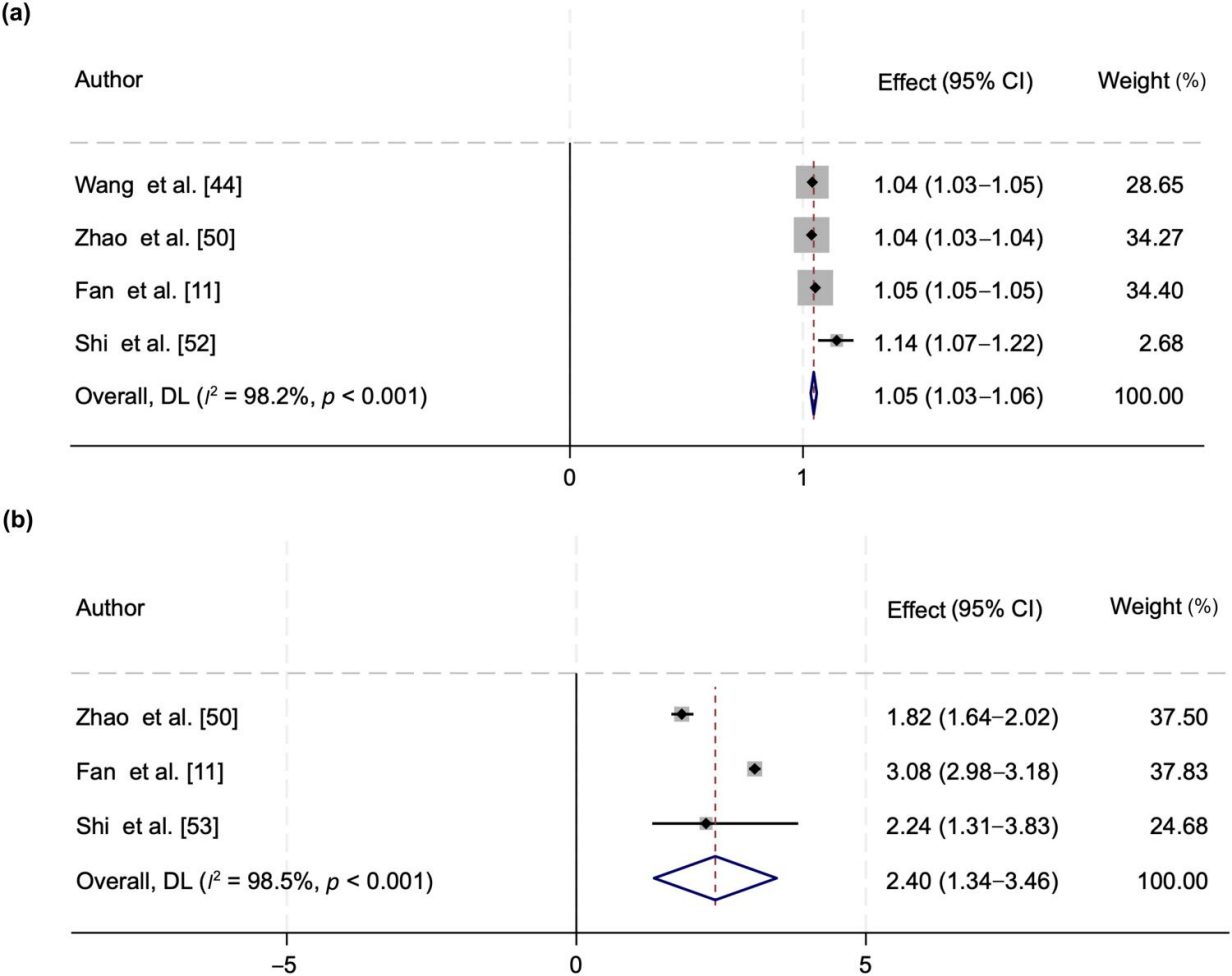
Meta‐analysis for HR of frailty for mortality. (a) The mortality risk of 1% increase in FI. (b) The mortality risk of frailty compared to robust. Weights are from random‐effects model. CI, confidence interval; DL, DerSimonian and Laird; HR, hazard ratio; FI, frailty index.

## 4 Discussion

This study synthesized evidence on frailty transitions, trajectories, and mortality among community‐dwelling Chinese adults. Across seven studies among Chinese adults aged 30 years and above with 2–11 years of follow‐up, 61% of participants remained stable frailty status, 29% worsened, and 10% improved. Distinct trajectories were identified, distinguished by either the rate of change or the level/change in FI. Frailty was associated with more than a twofold higher mortality risk compared with robust counterparts.

This review found that frailty progression may be preventable or modifiable. A meta‐analysis of 16 cohorts from different countries reported similar patterns, with 56.5% stable, 29.1% worsened, and 13.7% improved [5]. While intrinsic capacity tends to decline with age, resilience and interventions may counter functional loss in robust or prefrail individuals [56]. Improvements may also reflect short‐term fluctuations, the insensitivity of frailty measures to subtle changes over short follow‐up periods and measurement variability, or regression to the mean. Nonetheless, evidence of recovery underscores the potential biological and clinical reversibility of frailty [5, 8, 9], although the mechanisms underlying heterogeneity in transitions remain unclear. Of note, high heterogeneity existed in the pooled results of the proportions of different transitions and other meta‐analyses studying frailty transitions [5]. This might be explained by the differences in frailty measurements, areas, age, follow‐up duration, and inclusion/exclusion criteria for the study populations.

The widespread stability and potential reversibility of frailty provide a basis for developing targeted interventions. A study explored the trajectories of participants during a 1‐year period before death and found that some individuals were resilient to higher but stable levels of frailty and around one‐quarter experienced rapid deterioration [57]. Extending the stable phase and delaying decline should therefore be a key goal. Identifying correlates and mechanisms of recovery could enable targeted strategies to maximize improvement. Targeted interventions could be taken accordingly to maximize potential for recovery. Although lifestyle factors, muscle strength training, protein supplementation, comprehensive geriatric assessment, and multifactorial interventions show promise in previous studies [58, 59, 60, 61, 62], their population‐level effectiveness remains uncertain. In the Chinese context, translating these insights into practice requires alignment with existing healthcare delivery system and Healthy China 2030 initiative, which emphasizes strengthening primary healthcare and healthy ageing [63]. For example, frailty screening could be embedded in the National Basic Public Health Services Programme and family doctor contract services. Brief tools such as the FRAIL scale, Clinical Frailty Scale, or simplified frailty phenotype may be feasible in community settings because they are low‐cost and quick to administer. Screening could be conducted annually for older adults, with more often reassessment for those with prefrailty, multimorbidity, recent hospitalization, or functional decline. Family doctors and trained public health workers could conduct initial screening and refer frail individuals for comprehensive assessment and tailored interventions. Additionally, potential changes to practice should also consider broader challenges related to healthcare costs, feasibility, workforce capacity, and system‐level integration. Notably, the lack of well‐designed clinical trials on frailty prevention and treatment among Chinese population, hinders evidence‐based interventions [3].

This review provided a new perspective considering frailty trajectories and subgroups of individuals following unique developmental trajectories [64]. While dichotomous classifications (rapid vs. slow increase in FI) suggest consistent progression, most individuals remained stable in a frailty state across time points. This discrepancy may reflect differences in analytical approaches, follow up time and number of time‐points modelled. Transition‐based studies typically categorize individuals into discrete frailty states (robust, pre‐frail, frail) and model transitions between these states over single follow‐up time points, potentially overlooking within‐state variation or longer‐term trends. In contrast, trajectory analyses treat frailty as a continuous construct, modelling changes in frailty over multiple time‐points and longer‐term follow‐up and capturing gradual decline. Additionally, trajectories were also defined by baseline level and change of FI, which may help identify vulnerable subgroups. For example, a previous study found individuals with lower baseline electric FI but a rapid rise had greater mortality risk than those with higher, but stable frailty [57]. These findings indicate that both absolute frailty level and progression rate carry prognostic value, underscoring the need to link trajectories to health outcomes and to intervene early to improve prevention and management.

Frailty was consistently associated with higher mortality risk. The pooled HR of 1.05 per 1% increase in FI in this study aligns with a previous meta‐analysis (HR 1.04 across 13 cohorts) [10]. Another meta‐analysis, including 24 studies using different measurements (FP and FI), reported an overall HR of 2.34 between frailty and mortality [65], which is also similar with this study. These findings suggest comparable mortality risks in Chinese and non‐Chinese populations. Subgroup analyses further showed that the HR of a 1% increase in FI was higher in the older population and studies with a follow‐up duration of ≥ 10 years. The stronger association observed in older populations may reflect reduced physiological reserve, and consequently wider variation in health, among older individuals [66]. Studies with longer follow‐up durations may have accumulated a greater number of mortality events, particularly in relatively younger cohorts with lower short‐term mortality risk, thereby improving statistical power to detect associations. However, previous evidence does not confirm age as a modifier and findings on follow‐up duration remain inconsistent [67].

This study has limitations. First, substantial heterogeneity was observed, warranting cautious interpretation despite the use of random‐effects models. Second, some subgroup analyses (e.g., rural settings, adults < 60 years, regional comparisons) could not be meta‐analyzed, leaving heterogeneity unexplained. Third, analyses were limited to community‐dwelling adults and may not generalize to institutionalized populations. Additionally, only one publication from each cohort was included to avoid double‐counting participants. Consequently, the pooled estimates were based on a relatively small number of independent studies, which may have limited the precision and robustness of the findings. Another limitation of this review is that no widely accepted quality assessment tool is currently available for trajectory studies. Therefore, an adapted Newcastle–Ottawa Scale was used to evaluate the overall methodological quality of the included studies. However, the limitations were balanced by the novelty in conducting the first systematic review and meta‐analysis on transition, trajectory, and mortality for frailty in the Chinese population, showing a complete picture by including studies using different frailty measurements, and presenting detailed heterogeneity across different studies. Future research should clarify the mechanisms underlying frailty transitions and trajectories, identify their determinants and prognostic implications, and test interventions to delay progression, particularly in China. Economic evaluations of frailty states and interventions are also needed to inform policy and resource allocation. Additionally, evidence synthesis among other population like institutional individuals are also recommended.

## 5 Conclusions

Frailty was associated with higher mortality among Chinese community‐dwelling adults, yet progression was not inevitable. Distinct subpopulations emerged based on FI levels and rates of change. Future research should identify factors influencing transitions and trajectories and clarify their prognostic implications to inform targeted interventions that may delay or reverse frailty progression.

## Author Contributions

**Dongfeng Tang:** conceptualization, methodology, data curation, formal analysis, funding acquisition, writing – original draft, writing – review and editing. **Canlong Wang:** conceptualization, formal analysis, writing – review and editing, data curation. **Katie Jane Sheehan:** conceptualization, writing – review and editing, supervision. **Julie Whitney:** conceptualization, writing – review and editing. **Aicha Goubar:** conceptualization, writing – review and editing, supervision. **Salma Ayis:** methodology, writing – review and editing. **Matthew DL O'Connell:** conceptualization, methodology, supervision, writing – review and editing, data curation.

## Ethics Statement

The authors have nothing to report.

## Consent

The authors have nothing to report.

## Conflicts of Interest

The authors declare no conflicts of interest.

## Supporting information


Supporting File

## Data Availability

The data that support the findings of this study are available from the corresponding author upon reasonable request.
