# Supplementary material for: Systematic Review and Meta‐Analysis of the Transitions, Trajectories, and Associations With Mortality of Frailty in China
Source: Health Care Sci. 2026 Nov 2:10.1002/hcs2.70112. Online ahead of print. doi: 10.1002/hcs2.70112 (PMC13727761; doi:10.1002/hcs2.70112)
Supplement: Supplementary file 1 [file HCS2-9999-0-s001.pdf]

Supplementary materials

Table of Contents

Data S1. Search strategy 3

Table S1. Quality assessment of included studies 5

Figure S1. Proportions of individuals with different frailty status transitions 7

Figure S2. Publication bias of studies reporting proportions of frailty transitions 8

Table S2. Subgroup analysis of proportions of frailty transition status 9

Figure S3. HR of frailty for mortality 10

Figure S4. Publication bias of studies reporting mortality HRs of a 1% increase in FI 11

Figure S5. Publication bias of studies reporting mortality HRs of being frailty compared to robust 12

Table S3. Subgroup analysis of HR of 1% increase in FI and frailty 13

Data S1. Search strategy

PubMed search strategy:

#1 “frailty”[Mesh]
#2 frail* OR frailty syndrome* OR pre-frail OR frailty index OR frail elderly OR asthenia OR accumulated deficit
#3 (#1 OR #2)
#4 “China”[Mesh]
#5 China OR Chinese Mainland OR Chinese
#6 (#4 OR #5)
#7 (#3 AND #6)
#8 “prevalence”[Mesh] OR “epidemiology” [Mesh] OR “cross-sectional studies”[Mesh] OR “incidence”[Mesh]
#9 prevalence OR epidemiology OR cross-sectional studies OR cross-sectional study OR cross-sectional analysis OR Cross-Sectional analyses OR cross-sectional survey OR disease frequency survey OR incidence
#10 (#8 OR #9)
#11 “disease progression”[Mesh]
#12 trajectory OR trajectories OR change OR transition OR progression OR recovery OR recoveries OR increase OR decrease OR worsening OR improving OR pattern OR profile OR trend
#13 (#11 OR #12)
#14 “mortality”[Mesh] OR “survival”[Mesh] OR “survival analysis”[Mesh] OR “survival rate”[Mesh] OR “Kaplan-Meier estimate”[Mesh]
#15 all-cause mortality OR mortality OR death OR survival OR survival rate OR survival analysis OR Kaplan-Meier estimate
#16 (#14 OR #15)
#17 (#7 AND #10) OR (#7 AND #13) OR (#7 AND #16)

Embase search strategy:

#1 frail* OR frailty syndrome* OR pre-frail OR frailty index OR frail elderly OR asthenia OR accumulated deficit
#2 China OR Chinese Mainland OR Chinese
#3 (#1 AND #2)
#4 prevalence OR epidemiology OR cross-sectional studies OR cross-sectional study OR cross-sectional analysis OR Cross-Sectional analyses OR cross-sectional survey OR disease frequency survey OR incidence
#5 trajectory OR trajectories OR change OR transition OR progression OR recovery OR increase OR decrease OR worsening OR improving OR pattern OR profile OR trend
#6 all-cause mortality OR mortality OR death OR survival OR survival rate OR survival analysis OR Kaplan-Meier estimate
#7 (#3 AND #4) OR (#3 AND #5) OR (#3 AND #6)

Web of Science search strategy:

#1 TS= (frail* OR frailty syndrome* OR pre-frail OR frailty index OR frail elderly OR asthenia OR accumulated deficit)
#2 TS= (China OR Chinese Mainland OR Chinese)
#3 (#1 AND #2)
#4 TS= (prevalence OR epidemiology OR cross-sectional studies OR cross-sectional study OR cross-sectional analysis OR Cross-Sectional analyses OR cross-sectional survey OR disease frequency survey OR incidence)
#5 TS= (trajectory OR trajectories OR change OR transition OR progression OR recovery OR increase OR decrease OR worsening OR improving OR pattern OR profile OR trend)
#6 TS= (all-cause mortality OR mortality OR death OR survival OR survival rate OR survival analysis OR Kaplan-Meier estimate)
#7 (#3 AND #4) OR (#3 AND #5) OR (#3 AND #6)

CINAHL search strategy:

#1 TI (frail* OR frailty syndrome* OR pre-frail OR frailty index OR frail elderly OR asthenia OR accumulated deficit)
#2 AB (frailty OR frail* OR frailness OR frailty syndrome* OR pre-frail OR frailty index OR frail elderly OR asthenia OR accumulated deficit)
#3 SU (frailty OR frail* OR frailness OR frailty syndrome* OR pre-frail OR frailty index OR frail elderly OR asthenia OR accumulated deficit)
#4 (#1 OR #2 OR #3)
#5 TI (China OR Chinese Mainland OR Chinese)
#6 AB (China OR Chinese Mainland OR Chinese)
#7 SU (China OR Chinese Mainland OR Chinese)
#8 (#5 OR #6 OR #7)
#9 (#4 AND #8)
#10 TI (prevalence OR epidemiology OR cross-sectional studies OR cross-sectional study OR cross-sectional analysis OR Cross-Sectional analyses OR cross-sectional survey OR disease frequency survey OR incidence)
#11 AB (prevalence OR epidemiology OR cross-sectional studies OR cross-sectional study OR cross-sectional analysis OR Cross-Sectional analyses OR cross-sectional survey OR disease frequency survey OR incidence)
#12 SU (prevalence OR epidemiology OR cross-sectional studies OR cross-sectional study OR cross-sectional analysis OR Cross-Sectional analyses OR cross-sectional survey OR disease frequency survey OR incidence)
#13 (#10 OR #11 OR #12)
#14 TI (trajectory OR trajectories OR change OR transition OR progression OR recovery OR increase OR decrease OR worsening OR improving OR pattern OR profile OR trend)
#15 AB (trajectory OR trajectories OR change OR transition OR progression OR recovery OR increase OR decrease OR worsening OR improving OR pattern OR profile OR trend)
#16 SU (trajectory OR trajectories OR change OR transition OR progression OR recovery OR increase OR decrease OR worsening OR improving OR pattern OR profile OR trend)
#17 (#14 OR #15 OR #16)
#18 TI(all-cause mortality OR mortality OR death OR survival OR survival rate OR survival analysis OR Kaplan-Meier estimate)
#19 AB(all-cause mortality OR mortality OR death OR survival OR survival rate OR survival analysis OR Kaplan-Meier estimate)

#20 SU(all-cause mortality OR mortality OR death OR survival OR survival rate OR survival analysis OR Kaplan-Meier estimate)
#21 (#18 OR #18 OR #20)
#22 (#9 AND #13) OR (#9 AND #17) OR (#9 AND #21)

PsychInfo search strategy:

#1 AB OR TI (frail* OR frailty syndrome* OR pre-frail OR frailty index OR frail elderly OR asthenia OR accumulated deficit)
#2 AB OR TI (China OR Chinese Mainland OR Chinese)
#3 (#1 AND #2)
#4 AB OR TI (prevalence OR epidemiology OR cross-sectional studies OR cross-sectional study OR cross-sectional analysis OR Cross-Sectional analyses OR cross-sectional survey OR disease frequency survey OR incidence)
#5 AB OR TI (trajectory OR trajectories OR change OR transition OR progression OR recovery OR increase OR decrease OR worsening OR improving OR pattern OR profile OR trend)
#6 AB OR TI (all-cause mortality OR mortality OR death OR survival OR survival rate OR survival analysis OR Kaplan-Meier estimate)
#7 (#3 AND #4) OR (#3 AND #5) OR (#3 AND #6)

CNKI search strategy:

SU= (衰弱 + 虚弱 + 衰弱综合征 + 衰弱指数) * (发病率 + 患病率 + 现患率 + 流行病学轨迹 + 变化 + 死亡 + 生存)

WANFANF data search strategy:

#1 题名或关键词:((“衰弱" or “虚弱" or “衰弱综合征" or “衰弱指数") and (“发病率" or “患病率" or “现患率")) or 摘要:((“衰弱" or “虚弱" or “衰弱综合征" or “衰弱指数") and (“发病率" or “患病率" or “现患率"))
#2 题名或关键词:((“衰弱" or “虚弱" or “衰弱综合征" or “衰弱指数") and (“轨迹" or 变化)) or 摘要:((“衰弱" or “虚弱" or “衰弱综合征" or “衰弱指数") and (“轨迹" or 变化))
#3 题名或关键词:((“衰弱" or “虚弱" or “衰弱综合征" or “衰弱指数") and (“死亡" or “生存")) or 摘要:((“衰弱" or “虚弱" or “衰弱综合征" or “衰弱指数") and (“死亡" or “生存"))
#4 (#1 or #2 or #3)

VIP Information search strategy:

M=((衰弱 OR 虚弱 OR 衰弱综合征 OR 衰弱指数) AND (发病率 OR 患病率 OR 现患率 OR 流行病学 OR 轨迹 OR 变化 OR 死亡 OR 生存)) OR R=((衰弱 OR 虚弱 OR 衰弱综合征 OR 衰弱指数) AND (发病率 OR 患病率 OR 现患率 OR 流行病学 OR 轨迹 OR 变化 OR 死亡 OR 生存))

Table S1. Quality assessment of included studies

Study	Representative	Selection of non frail comparison	Ascertainment of exposure frailty	Non respondents	Outcome not present at start	Controls for age and sex	Controls for other factors	Outcome assessment	Length of follow up	Adequacy of follow up	Score of trajectory studies	Score of mortality studies
Wang, et al. [20]	1	.	1	0	.	.	.	.	.	.	2	.
Duan, et al. [21]	1	.	2	0	.	.	.	.	.	.	3	.
Jia, et al. [22]	1	.	2	0	.	.	.	.	.	.	3	.
Ye, et al. [23]	0	.	2	1	.	.	.	.	.	.	3	.
He, et al. [24]	0	.	2	0	.	.	.	.	.	.	2	.
Bi, et al. [25]	1	.	2	0	.	.	.	.	.	.	3	.
Yang, et al. [26]	1	.	2	0	.	.	.	.	.	.	3	.
Lin, et al. [27]	1	.	2	0	.	.	.	.	.	.	3	.
Yang, et al. [28]	0	.	2	0	.	.	.	.	.	.	2	.
Du, et al. [29]	1	.	2	0	.	.	.	.	.	.	3	.
Guo, et al. [30]	1	.	2	1	.	.	.	.	.	.	4	.
Guo, et al. [31]	1	.	2	0	.	.	.	.	.	.	3	.
Liu, et al. [32]	1	.	2	1	.	.	.	.	.	.	4	.
Chu, et al. [33]	1	.	2	0	.	.	.	.	.	.	3	.
Wu, et al. [34]	1		2	1							4	
Sha, et al. [35]	1	.	2	1	.	.	.	.	.	.	4	.
Zhu, et al. [36]	1	.	2	0	.	.	.	.	.	.	3	.
Pan, et al. [37]	1	.	2	0	.	.	.	.	.	.	3	.
Sha, et al. [38]	1	.	2	0	.	.	.	.	.	.	3	.
Sheng, et al. [39]	1	.	2	0	.	.	.	.	.	.	3	.
Liu, et al. [40]	0	.	2	0	.	.	.	.	.	.	2	.
Fan, et al. [41]	0	.	2	0	.	.	.	.	.	.	2	.
Li, et al. [42]	1	.	2	0	.	.	.	.	.	.	3	.
Chen [43]	1	.	2	0	.	.	.	.	.	.	3	.
Wang, et al. [44]	1	1	2	0	1	1	1	1	1	1	.	10

Shi, et al. [45]	0	1	2	1	1	1	1	1	1	1	.	10
Cao, et al. [46]	1	1	2	0	1	1	1	1	1	0	.	9
Sun, et al. [47]	1	1	2	0	1	1	1	1	1	0	.	9
Wu, et al. [48]	1	1	2	0	1	1	1	1	1	0	.	9
Fan, et al. [49]	1	1	2	0	1	0	1	1	0	0	.	7
Fan, et al. [11]	1	1	2	0	1	1	1	1	1	0	.	9
Zhao, et al. [50]	1	1	2	0	1	1	1	1	1	0	.	9
She, et al. [51]	1	1	2	0	1	1	1	1	1	1	.	10
Shi, et al. [52]	0	1	2	1	1	1	1	1	1	1	.	10
Shi, et al. [53]	0	1	2	0	1	1	1	1	1	1	.	9
Gu, et al. [54]	1	1	2	1	1	1	1	1	1	1	.	11
Sun, et al. [55]	0	1	2	0	1	0	1	1	1	0	.	7

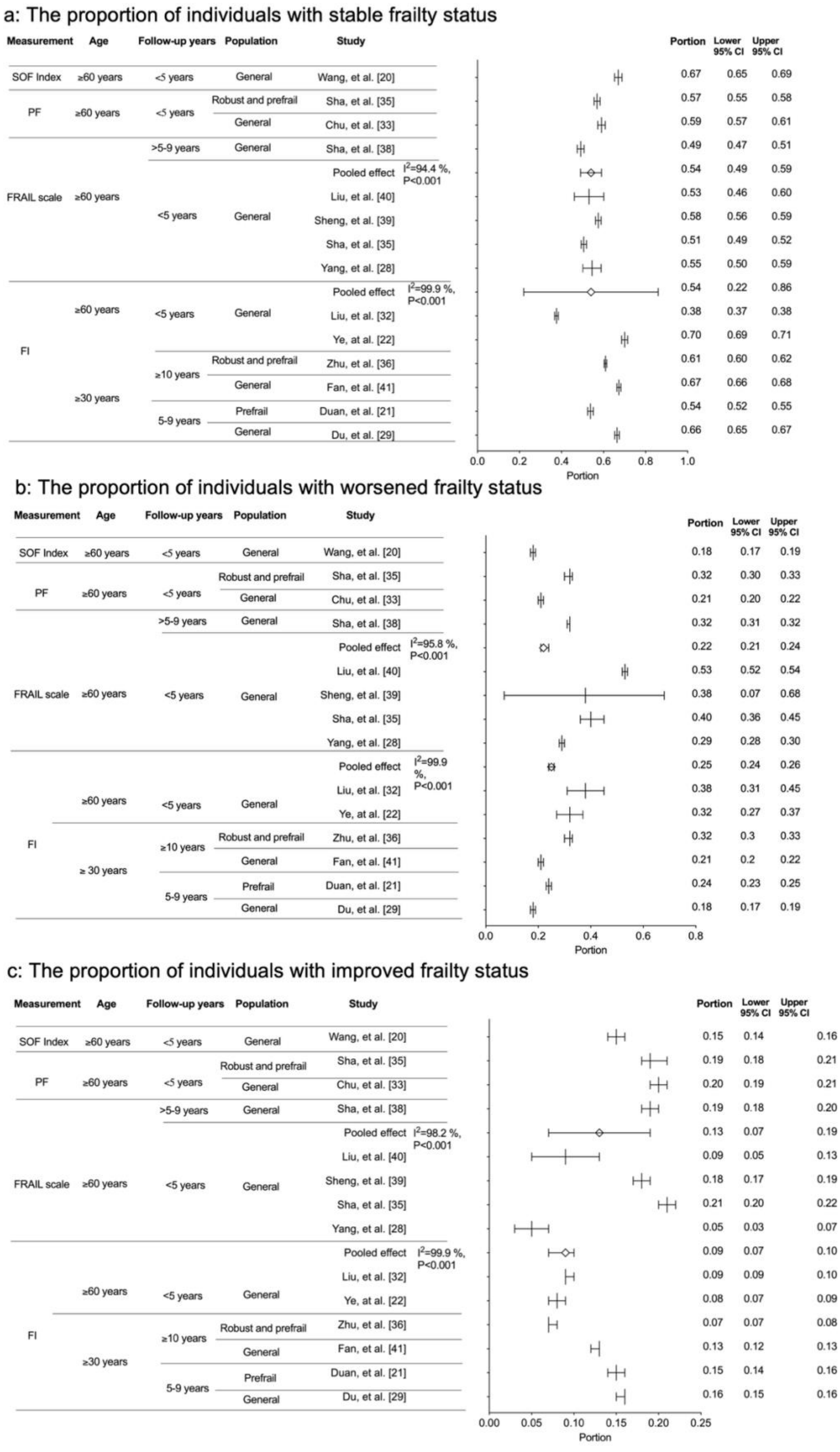

Figure S1. Proportions of individuals with different frailty status transitions

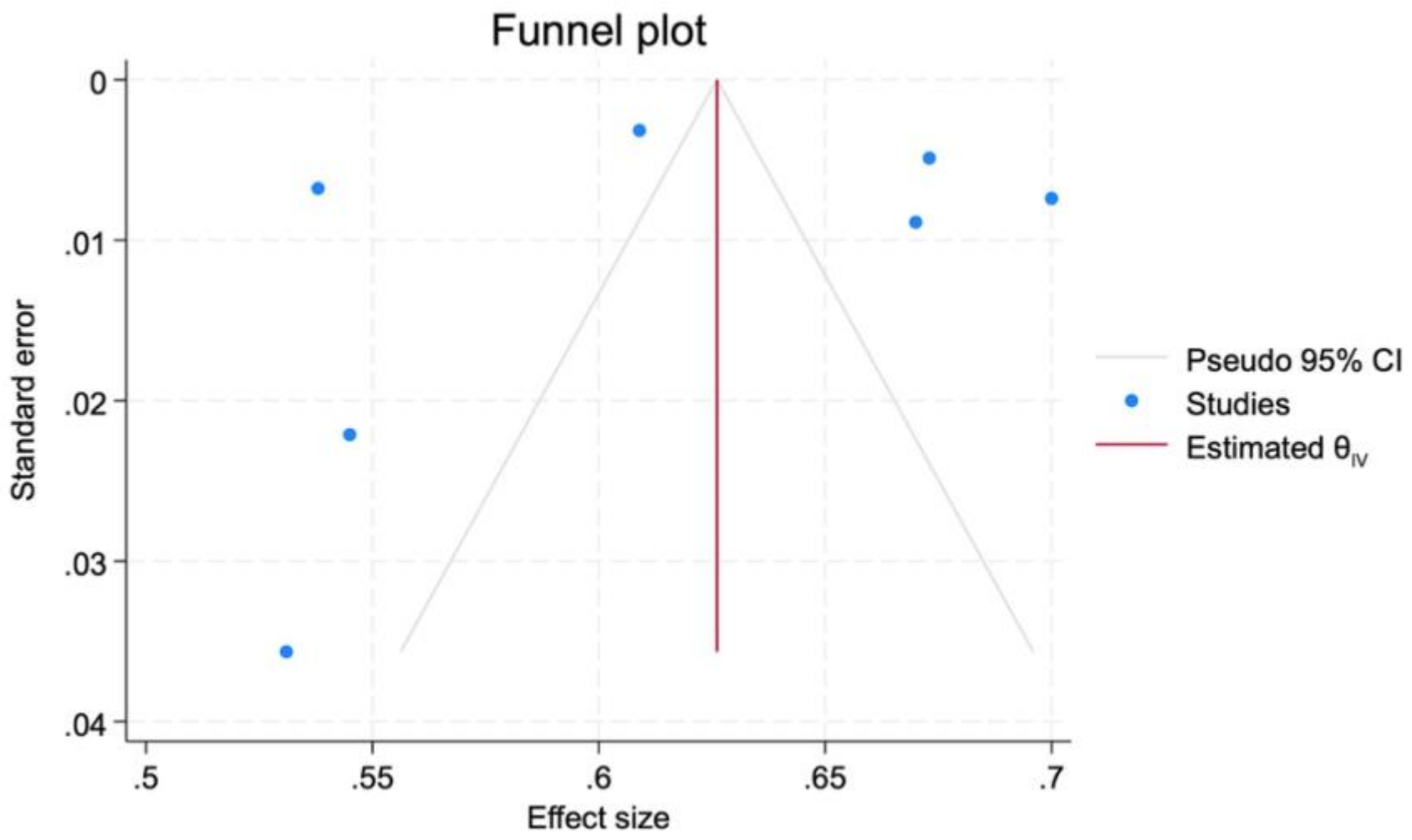

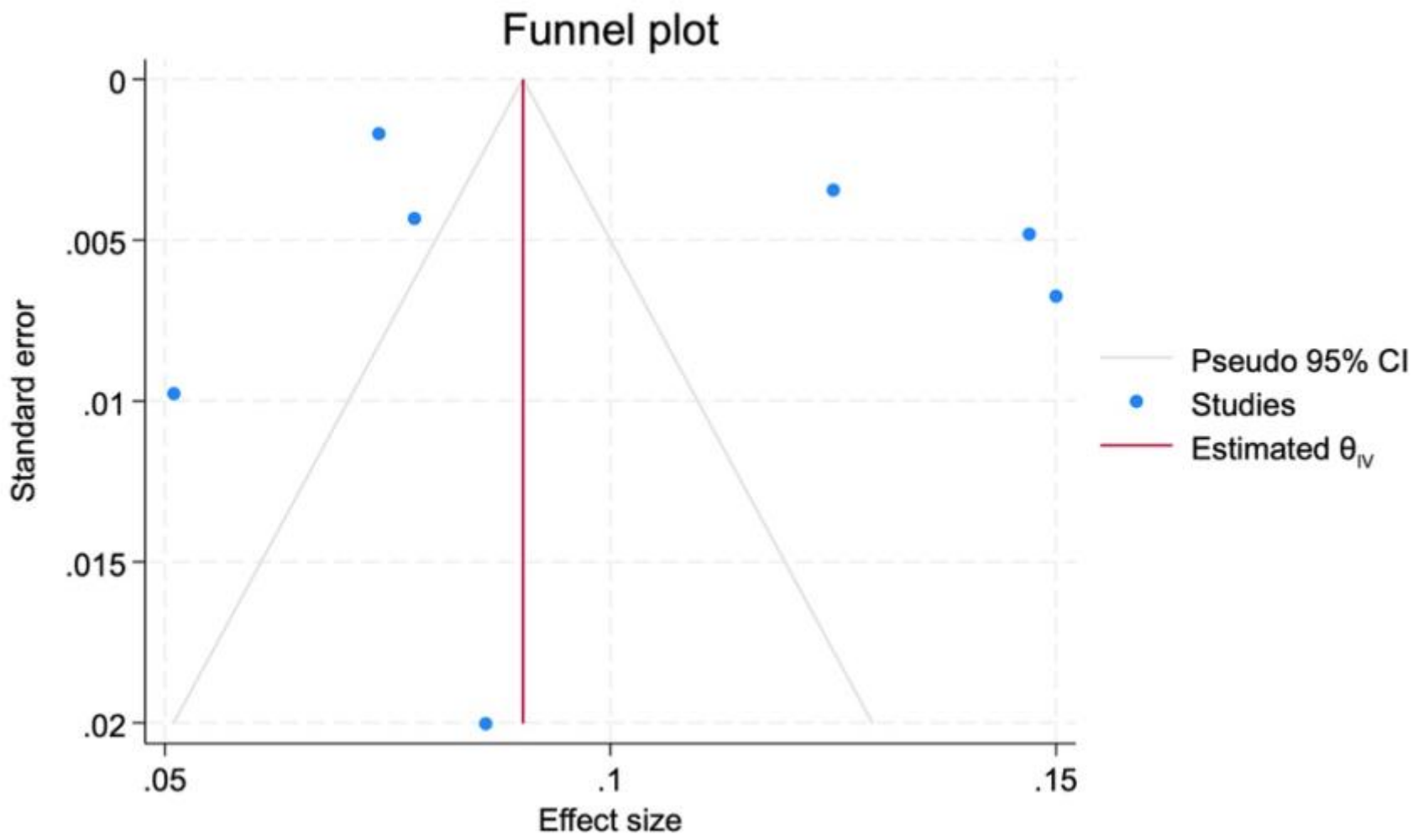

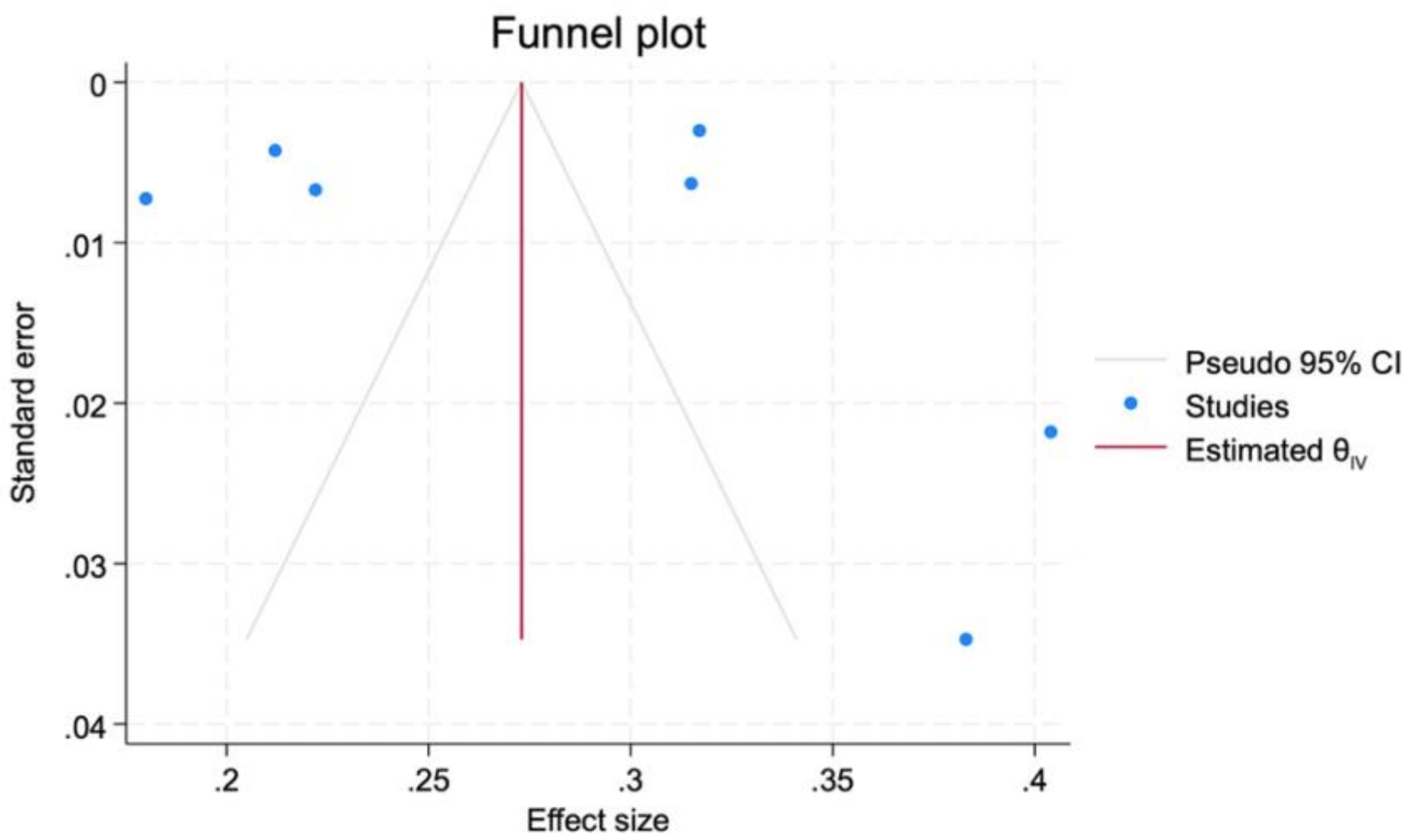

Figure S2. Publication bias of studies reporting proportions of frailty transitions

Table S2. Subgroup analysis of proportions of frailty transition status

Subgroup	Study number	Stable		Worsen		Improve	
		Pooled proportions	Heterogeneity	Pooled proportions	Heterogeneity	Pooled proportions	Heterogeneity
Overall	7	0·61 (0·57-0·66)	I^2=98·6%, P<0·001	0·29 (0·24-0·34)	I^2=99·2%, P<0·001	0·10 (0·07-0·13)	I^2=98·6%, P<0·001
Measurement							
FI	5	0·58 (0·47-0·69)	I^2=99·9%, P<0·001	0·32 (0·21-0·43)	I^2=99·9%, P<0·001	0·10 (0·08-0·13)	I^2=98·8%, P<0·001
FRAIL	4	0·54 (0·48-0·59)	I^2=95·1%, P<0·001	0·33 (0·27-0·40)	I^2=96·9%, P<0·001	0·13 (0·07-0·19)	I^2=98·3%, P<0·001
Area							
Urban	3	0·59 (0·48-0·69)	I^2=95·7%, P<0·001	0·33 (0·18-0·48)	I^2=97·9%, P<0·001	0·09 (0·03-0·14)	I^2=96·2%, P<0·001
Age							
≥60 years	5	0·61 (0·56-0·67)	I^2=96·9%, P<0·001	0·27 (0·22-0·32)	I^2=96·8%, P<0·001	0·11 (0·06-0·17)	I^2=98·6%, P<0·001
Follow-up duration							
<5 years	5	0·61 (0·56-0·67)	I^2=96·9%, P<0·001	0·27 (0·22-0·32)	I^2=96·8%, P<0·001	0·11 (0·06-0·17)	I^2=98·6%, P<0·001
≥10 years	2	0·64 (0·58-0·70)	I^2=99·2%, P<0·001	0·26 (0·16-0·37)	I^2=99·8%, P<0·001	0·10 (0·05-0·15)	I^2=99·4%, P<0·001

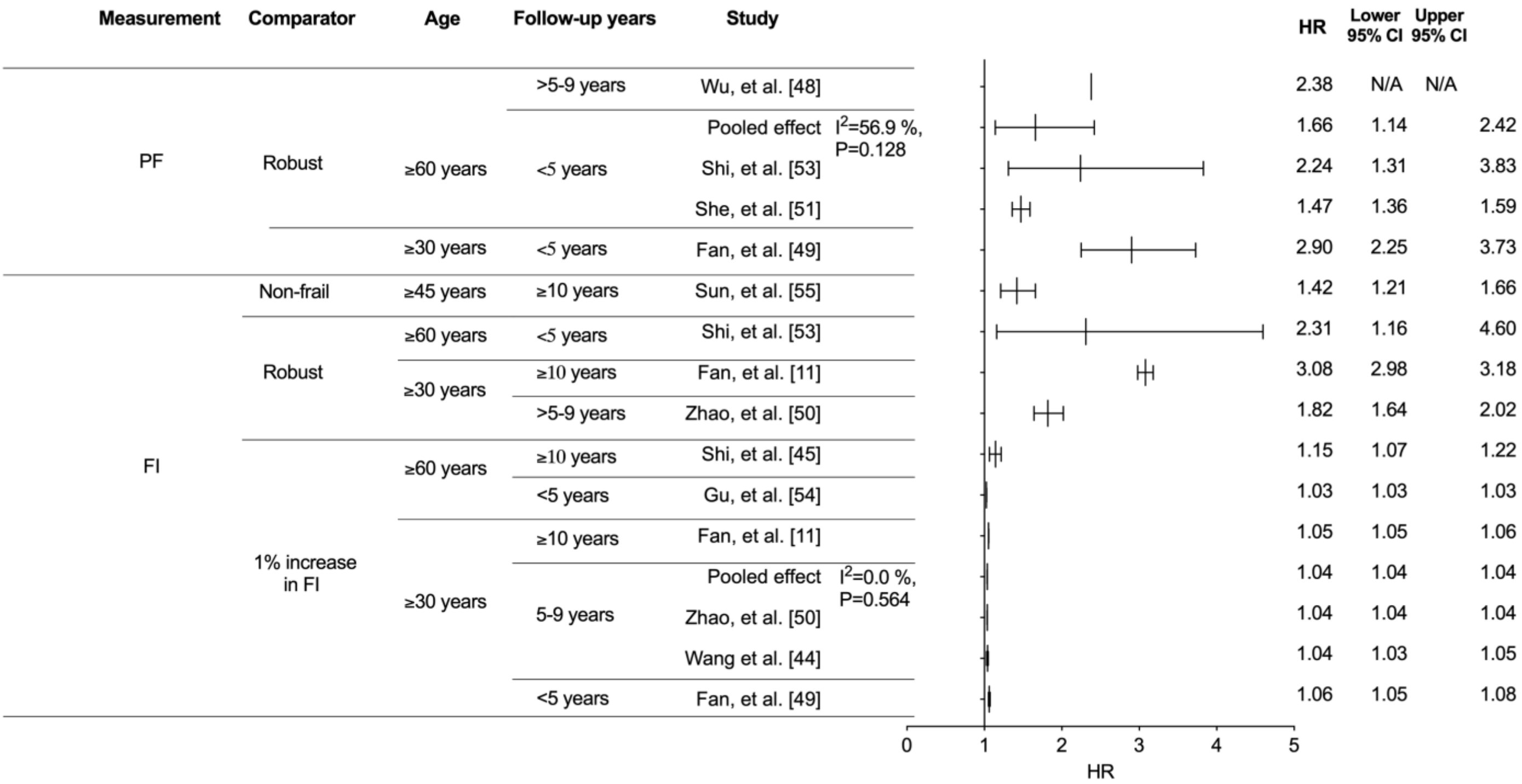

Figure S3. HR of frailty for mortality

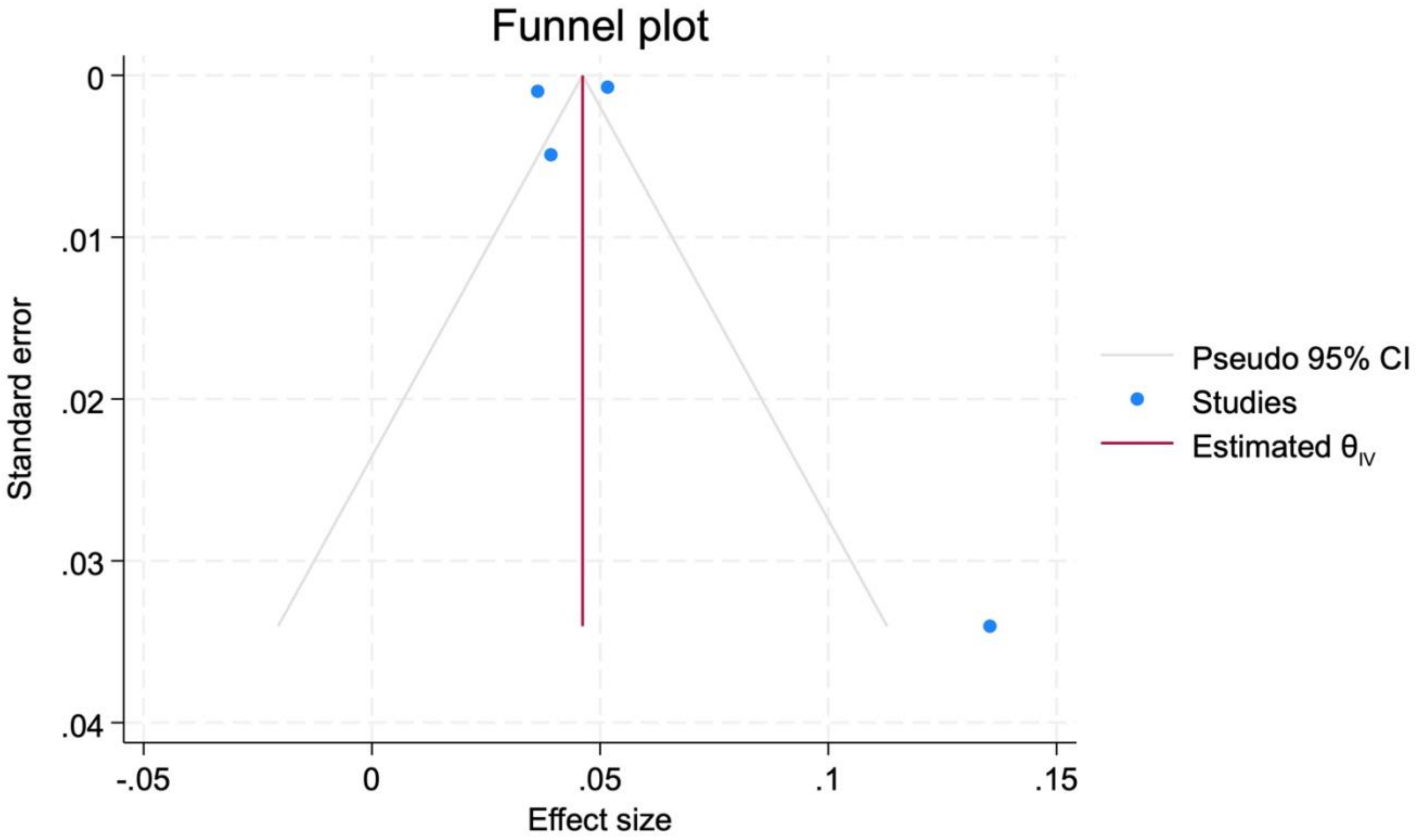

Figure S4. Publication bias of studies reporting mortality HRs of a 1% increase in FI

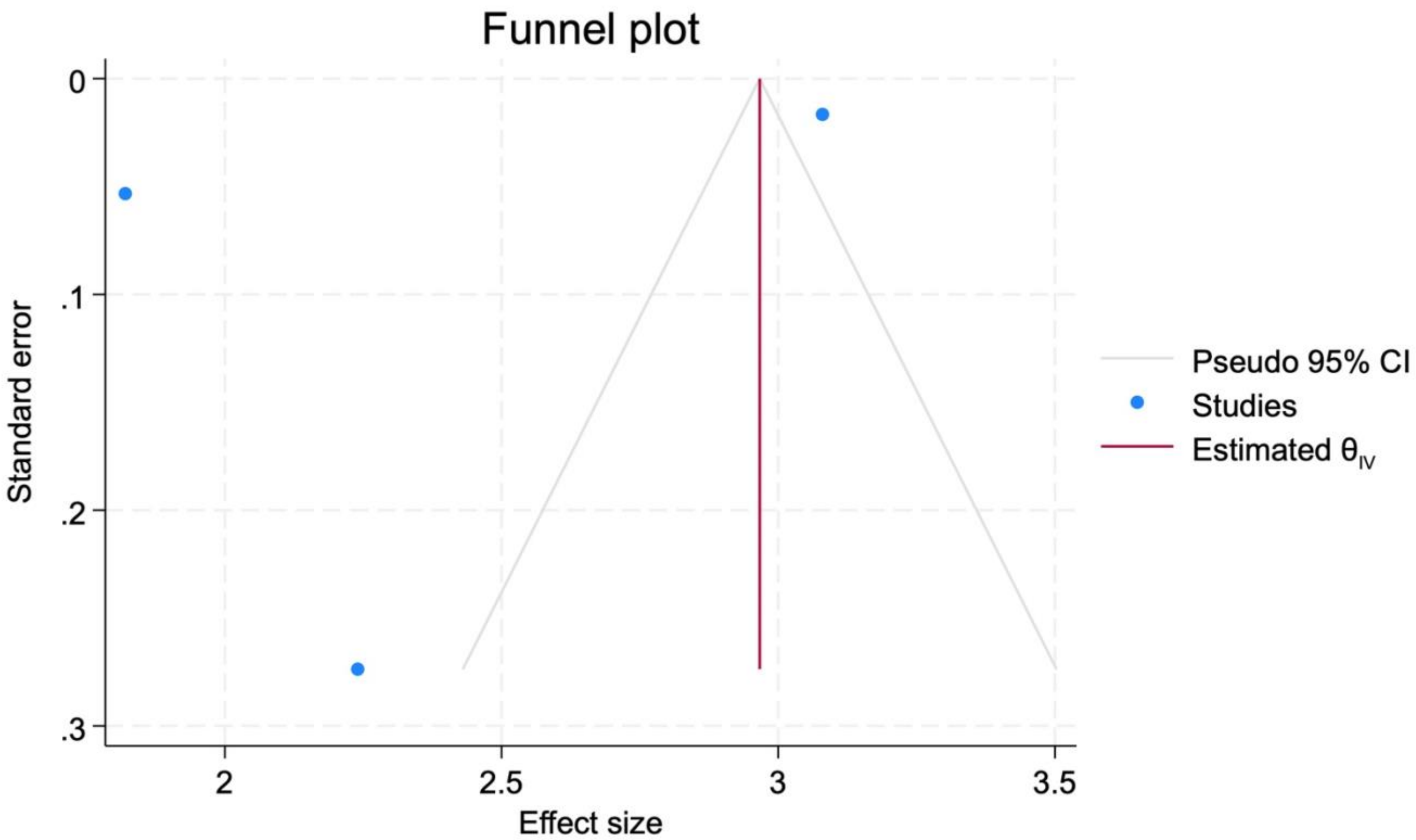

Figure S5. Publication bias of studies reporting mortality HRs of being frailty compared to robust

Table S3. Subgroup analysis of HR of 1% increase in FI and frailty

Subgroup	1% increase in FI			Frailty		
	Study number	HR (95% CI)	Heterogeneity	Study number	HR (95%CI)	Heterogeneity
Overall	4	1·05 (1·03-1·06)	I^2=98·2%, p<0·001	3	2·34 (1·51-3·63)	I^2=97·8%, p<0·001
Measurement						
FI	/			3	2·36 (1·50-3·71)	I^2=97·8%, p<0·001
PF				3	2·09 (1·24-3·53)	I^2=92·6%, p<0·001
Area						
Urban	1	1·15 (1·07–1·22)	/	/		
Rural	/			1	2·24 (1·31-3·83)	/
Age						
≥60 years	2	1·08 (0·97-1·20)	I^2=90·2%, p=0·001	2	1·66 (1·14-2·42)	I^2=56·9%, p=0·128
Follow-up duration						
<5 years	2	1·04 (1·01-1·08)	I^2=94·7%, p<0·001	3	2·09 (1·24-3·53)	I^2=92·6%, p<0·001
5-9 years	2	1·04 (1·04-1·04)	I^2=0·00%, p=0·564	1	1·82 (1·64-2·02)	/
≥10 years	2	1·09 (1·01-1·18)	I^2=83·5%, p=0·014	1	3·08 (2·98-3·18)	/
